# Partner‐ and Mode‐Specific Social Contact and Poor Mental Health Among Older Adults in Japan: A Cross‐Sectional Analysis of the 2024 JACSIS Study

**DOI:** 10.1111/ggi.70840

**Published:** 2026-09-13

**Authors:** Kazumi Kubota, Miya Aishima, Yukiko Matsumura, Takeshi Miura, Takahiro Tabuchi

**Affiliations:** ^1^ Shimonoseki City University Shimonoseki Japan; ^2^ Department of Healthcare Information Management The University of Tokyo Hospital Tokyo Japan; ^3^ National Center for Child Health and Development Tokyo Japan; ^4^ Department of Nursing Shimonoseki City University Shimonoseki Japan; ^5^ Department of Gerontological Nursing, School of Medicine Yokohama City University Yokohama Japan; ^6^ Division of Epidemiology, School of Public Health Tohoku University Graduate School of Medicine Sendai Japan

**Keywords:** in‐person contact, Japan, mental health, older adults, social relationships

## Abstract

**Aim:**

To examine whether social contact frequency is differentially associated with poor mental health by interaction partner and contact mode among older adults in Japan.

**Methods:**

We analyzed the 2024 Japan COVID‐19 and Society Internet Survey (JACSIS; December 2024–January 2025). All respondents aged ≥ 65 years in the analysis‐ready dataset were included; no analytic variables had missing values. Poor mental health was defined as ≥ 1 versus 0 poor mental health days in the past 30 days (CDC Healthy Days item). Eight past‐month partner–mode exposures (family or friends by in‐person, phone, email/messages, or video call) were categorized as none, 1–3 times/month, or ≥ 1 time/week. Logistic regression estimated adjusted odds ratios (ORs) and 95% confidence intervals (CIs), adjusting for demographic, health, lifestyle, physical activity, employment, education, and household income. False discovery rate control was applied across 16 prespecified contrasts.

**Results:**

Among 6786 older adults (mean age 72.7 years; 50.4% men), 11.8% reported ≥ 1 poor mental health day. In fully adjusted models, more frequent in‐person contact was associated with lower odds of poor mental health for friends/acquaintances (vs none: 1–3/month OR 0.71, 95% CI 0.59–0.84; ≥ 1/week 0.59, 0.48–0.73) and non‐cohabiting family/relatives (0.84, 0.71–0.99; 0.71, 0.57–0.89). Remote modes were not clearly associated. After multiplicity control, weekly in‐person family contact and both friend in‐person frequencies remained significant.

**Conclusions:**

Frequent in‐person contact, especially with friends, was associated with lower odds of poor mental health, whereas most remote modes were not, even after adjustment for education and household income.

## Graphical abstract

In this nationwide web‐based study of older adults in Japan, frequent face‐to‐face contact, especially with friends, was associated with lower odds of poor mental health. Most remote contact modes showed weaker or inconsistent associations, suggesting that maintaining in‐person social engagement may be important for mental well‐being in later life.

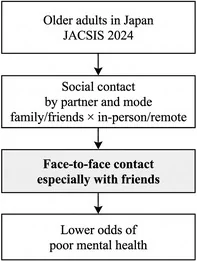

## 1 Introduction

Maintaining functional ability and well‐being in later life is central to healthy aging. Social relationships may support these outcomes through emotional support, companionship, perceived belonging, behavioral activation, maintenance of daily routines, and access to practical assistance. Consistent with social integration and social support frameworks, stronger social ties have been linked to lower mortality and better mental health across diverse settings [1, 2, 3, 4, 5, 6]. Social isolation and loneliness among older adults are increasingly prioritized as public health challenges with potential solutions in health care and community systems [7, 8]. In Japan—one of the most rapidly aging societies globally [9]—evidence on everyday social behaviors that relate to mental well‐being has direct relevance for geriatric practice, prevention, and community design.

Social isolation and loneliness frameworks further suggest that insufficient or unsatisfying social connections can contribute to psychological distress in later life. From an active aging perspective, meaningful social participation is also important for autonomy, function, and well‐being among older adults. Therefore, distinguishing the type of social contact that is most closely related to mental health may have practical relevance for geriatric care and community‐based prevention.

Two practical gaps remain. First, the mode of contact may matter. Older adults communicate face‐to‐face and through remote modalities such as phone calls, email/messages, and video calls. Since the COVID‐19 pandemic, remote communication has become more common, but reviews of technology‐mediated interventions in older adults suggest heterogeneous and often modest effects on loneliness and mental health, implying that remote contact may not function as a simple substitute for in‐person engagement [10, 11, 12]. Second, the interaction partner may matter. Family and friends can play distinct roles in providing support, companionship, identity, and opportunities for shared activities, with potentially different implications for mental health [1, 2, 13, 14, 15]. Friend contact may reflect discretionary participation and shared activities that support positive affect and purpose in later life [14, 15]. A US cohort study reported that face‐to‐face, but not phone or email, contact predicted lower subsequent depression in older adults [16], underscoring the need to jointly consider partner and mode.

Using nationwide data from the 2024 Japan COVID‐19 and Society Internet Survey (JACSIS), we addressed two research questions: first, whether more frequent social contact was associated with lower odds of poor mental health among older adults in Japan; and second, whether these associations differed by interaction partner and contact mode. We focused on non‐cohabiting family/relatives and friends/acquaintances, and on in‐person, phone/audio‐only, email/messages, and video contact. We hypothesized that in‐person contact would show stronger inverse associations with poor mental health than remote contact, and that associations would be particularly evident for contact with friends/acquaintances because such contact may reflect discretionary social participation and shared activities [14, 15]. Because multiple contrasts were examined, we prespecified false discovery rate control across primary comparisons [17].

## 2 Methods

### 2.1 Study Design and Setting

This was a cross‐sectional analysis of the 2024 Japan COVID‐19 and Society Internet Survey (JACSIS), an ongoing, large‐scale, web‐based, self‐administered questionnaire survey that monitors health, social, behavioral, and socioeconomic conditions in Japan [18]. The 2024 wave was conducted from 2 December 2024 to 16 January 2025 and collected information on participants' demographic characteristics, lifestyle factors, health status, social relationships, and socioeconomic circumstances. Participants were recruited from a large commercial online survey panel administered by Rakuten Insight Inc. (Tokyo, Japan). Sampling was designed to reflect the distributions of sex, age groups, and residential regions across all 47 prefectures in Japan. Eligible respondents were adults living in Japan who were registered with the survey panel and were invited to participate by the survey company. Participants completed the questionnaire themselves online after providing electronic informed consent. However, because the source panel was a commercial web survey panel, it did not constitute a strict probability sample. The primary analyses were conducted without applying additional individual sampling weights. The 2024 JACSIS dataset used in this study was provided to the authors as an analysis‐ready dataset after survey allocation and JACSIS quality‐control procedures. Because the present analysis focused on the older‐adult subset of this dataset, and because individual‐level sampling weights appropriate for weighted regression within this specific analytic subset were not available, additional sampling weights were not applied. Accordingly, the findings should be interpreted as associations observed within the analytic sample, rather than as nationally representative prevalence estimates for the Japanese older‐adult population.

### 2.2 Participants

**FIGURE 1:**
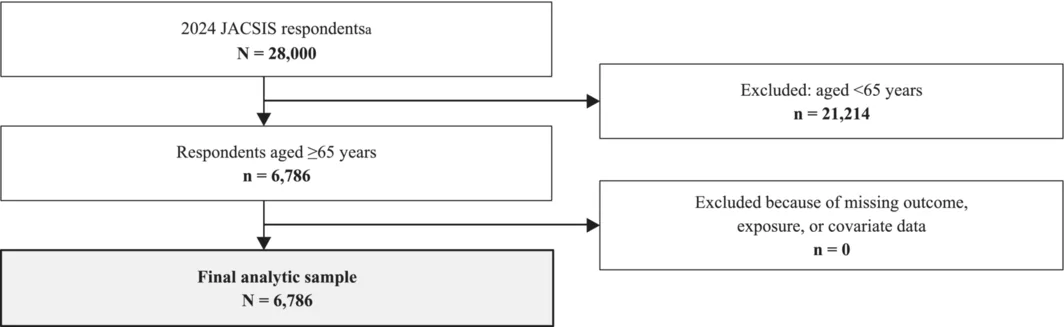
Participant selection flow within the analysis‐ready 2024 JACSIS dataset. ^a^ The 2024 JACSIS dataset was provided to the authors as an analysis‐ready dataset after survey allocation and the JACSIS quality‐control procedures, which comprise attention‐check items and checks for inconsistent or implausible responses. The number of panel members invited to participate, the number of completed responses before quality control, the corresponding response rate, and the number of respondents excluded by the quality‐control procedures were not included in the analysis‐ready dataset available to the authors. *Note:* JACSIS, Japan COVID‐19 and Society Internet Survey. The 2024 wave was fielded from 2 December 2024 to 16 January 2025. Covariate data comprise age, sex, household size, morbidity, smoking, drinking, walking, running/jogging, sport, working status, education, and household income. The findings should be interpreted as associations within the analytic sample rather than nationally representative prevalence estimates.

The 2024 JACSIS dataset used in this study was provided to the authors as an analysis‐ready dataset comprising 28 000 respondents, after survey allocation and the JACSIS quality‐control procedures. Upstream panel‐management information, including the number of panel members invited to participate, the number completing the survey before quality control, the corresponding response rate, and the number of respondents excluded by the quality‐control procedures, was not included in the analysis‐ready dataset available to the authors; these figures were managed by the survey company and the JACSIS operations team. For the present analysis, we restricted the sample to adults aged 65 years or older. Among the 28 000 analysis‐ready respondents, 6786 met this age criterion. All 6786 eligible older respondents had complete responses for the outcome, exposure variables, education, household income, and all other covariates used in the present analysis; therefore, no respondents aged ≥ 65 years were excluded because of missing analytic variables. The final analytic sample comprised 6786 older adults. The participant selection process available within the analysis‐ready dataset is shown in Figure 1.

To improve data quality, the JACSIS study incorporated procedures to identify potentially unreliable responses, including attention‐check items and checks for inconsistent or implausible answers. Respondents identified as satisficers according to the JACSIS quality‐control criteria were excluded before the analysis‐ready dataset was provided.

### 2.3 Outcome: Poor Mental Health

Poor mental health was assessed using the CDC Healthy Days mental health item: “During the past 30 days, for about how many days was your mental health not good?” [19, 20]. The Healthy Days measures have been widely used for population health surveillance, and the Japanese version of the CDC HRQOL‐4 has shown acceptable reliability and validity [21]. Responses range from 0 to 30 days. For the primary analysis, responses were dichotomised as 0 versus ≥ 1 day because the distribution was highly concentrated at zero. We interpreted this outcome as an indicator of any recent poor mental health, not as a clinical diagnosis or a screening threshold for depressive or anxiety disorders. The conventional threshold of ≥ 14 mentally unhealthy days is often used to define frequent mental distress; however, this was not used as the primary outcome because the present study aimed to capture any recent poor mental health in this older‐adult sample.

### 2.4 Exposures: Frequency of Social Contact by Partner and Mode

Eight exposures were created by crossing interaction partner with contact mode. Participants were asked how often they had contact during the past month with each type of interaction partner by each communication mode. Partners were non‐cohabiting family/relatives and friends/acquaintances. Modes were in‐person contact, phone/audio‐only contact, email/messages including chat applications such as LINE, and video calls. For each partner–mode combination, response options were none, once/month, two‐three times/month, once/week, two‐three times/week, four‐five times/week, and almost daily. These were recoded into none, 1–3 times/month, or ≥ 1 time/week.

### 2.5 Covariates

Based on social epidemiology frameworks linking social ties to health [1, 13], covariates were selected a priori to adjust for major demographic, household, health, socioeconomic, lifestyle, physical activity, and employment‐related factors while maintaining a parsimonious model across the eight partner–mode exposure variables. We included age, sex, household size, morbidity, working status, education, and household income. Household size was included to account for immediate household context, and working status, education, and household income were included as employment‐related and socioeconomic factors.

Morbidity was coded as present if respondents reported either a past diagnosis or any current condition for at least one of the following: diabetes; pneumonia/bronchitis; asthma; atopic dermatitis; ischaemic heart disease or myocardial infarction; stroke; chronic obstructive pulmonary disease; chronic kidney disease; chronic hepatitis/cirrhosis; cancer; epilepsy; depression; or other mental disorder. We did not exclude respondents with depression or other mental disorders because these conditions are part of the health profile of older adults and exclusion could reduce generalizability. Although K6 psychological distress scores were available in the survey, K6 was not included as a covariate because it conceptually overlaps with the outcome of poor mental health. The coding of morbidity was independently verified by two researchers.

Lifestyle covariates included smoking, drinking, and physical activity indicators. Smoking was coded as ever versus never, drinking as any versus never, and walking, running/jogging, and sport were each coded yes if average daily time was greater than zero. Socioeconomic covariates included education and household income. Each was entered as a binary indicator: education as junior college or lower versus all other responses, and household income as < 4 million JPY versus all other responses. Marital status was not included in the primary model because the main objective was to compare associations across partner–mode contact categories using a consistent prespecified adjustment set, and because marital status may overlap conceptually with household structure and with the availability or meaning of family contact. Possible residual confounding by marital context was therefore considered in the interpretation.

### 2.6 Statistical Analysis

For each of the eight exposures, we fitted separate logistic regression models estimating odds ratios and 95% confidence intervals for 1–3 times/month and ≥ 1 time/week compared with none. Model 1 was unadjusted. Model 2 adjusted for age, sex, household size, and morbidity. Model 3 additionally adjusted for smoking, drinking, walking, running/jogging, sport, working status, education, and household income. In Model 3, we tested linear trend across ordered exposure categories by scoring none, 1–3 times/month, and ≥ 1 time/week as 0, 1, and 2, respectively. To address multiplicity across 16 prespecified primary contrasts, we controlled the false discovery rate using the Benjamini–Hochberg procedure [17].

The survey was conducted using a required‐response format, in which participants were required to provide a response to all items before proceeding; therefore, no missing values were generated for individual variables [22]. Accordingly, there were no missing values in the analytic variables among eligible older respondents, including education and household income, and no imputation or listwise deletion was required. We did not conduct extensive post hoc interaction analyses because the study examined eight partner–mode exposures and 16 prespecified primary contrasts, and multiple exploratory interaction models would substantially increase model complexity and multiplicity. Instead, we interpreted the findings in relation to household and social context and acknowledged this issue as a limitation. Two‐sided *p* values below 0.05 were considered statistically significant. Analyses were performed using IBM SPSS Statistics version 29.

### 2.7 Ethics

The study used de‐identified data. Ethical approval was obtained from the Shimonoseki City University Research Ethics Committee (acceptance no. 2509‐0623) and the Tohoku University Graduate School of Medicine Ethics Committee (rapid review dated 26 March 2025; study number 2024‐1‐1035). Participants provided electronic informed consent. The study was conducted in accordance with the Declaration of Helsinki and relevant institutional guidelines and regulations.

## 3 Results

### 3.1 Demographic Characteristics and Exposure Frequencies

The participant selection process is shown in Figure 1. Among 6786 older adults, the mean age was 72.7 years and 50.4% were men. Overall, 11.8% reported at least one poor mental health day in the prior 30 days (Table 1). In‐person contact was common: for non‐cohabiting family/relatives, 45.3% reported 1–3 times/month and 18.7% ≥ 1 time/week; for friends/acquaintances, 43.1% and 27.5% reported these frequencies, respectively. Remote contact was generally less frequent, particularly video calls, and distributions for all eight exposures are shown in Table S1.

**TABLE 1:** Demographic characteristics of this study.

Characteristic	Overall (*N* = 6786)	No poor mental health days (0 days) (*n* = 5983; 88.2%)	Poor mental health days (≥ 1 day) (*n* = 803; 11.8%)
Demographic characteristics			
Age, mean (SD), years	72.7 (4.9)	72.7 (4.9)	72.4 (5.0)
Sex, male, *n* (%)	3423 (50.4)	3116 (52.1)	307 (38.2)
Household size, mean (SD)	2.08 (0.77)	2.10 (0.76)	1.98 (0.83)
Morbidity (past and/or current), *n* (%)	2921 (43.0)	2680 (44.8)	241 (30.0)
Socioeconomic characteristics			
Education ≤ junior college, *n* (%)	3788 (55.8)	3291 (55.0)	497 (61.9)
Household income < 4 million JPY, *n* (%)	3017 (44.5)	2607 (43.6)	410 (51.1)
Lifestyle, exercise, and employment			
Smoking habit (Yes), *n* (%)	3393 (50.0)	2997 (50.1)	396 (49.3)
Drinking habit (Yes), *n* (%)	3121 (46.0)	2723 (45.5)	398 (49.6)
Walking (exercise) habit (Yes), *n* (%)	292 (4.3)	250 (4.2)	42 (5.2)
Running/jogging habit (Yes), *n* (%)	1039 (15.3)	915 (15.3)	124 (15.4)
Sports habit (Yes), *n* (%)	4513 (66.5)	3941 (65.9)	572 (71.2)
Working status (Yes), *n* (%)	1854 (27.3)	1646 (27.5)	208 (25.9)

*Note:* Values are presented as *n* (%) unless otherwise indicated. Values are unweighted; percentages may not sum to 100% due to rounding. Exposure distributions are presented in Table S1. Morbidity was defined as reporting at least one physician‐diagnosed condition either in the past or currently, including depression or other mental disorder, as described in Methods; morbidity coding was confirmed by two researchers. Education and household income were each analyzed as a binary indicator: junior college or lower versus all other responses, and < 4 million JPY versus all other responses, respectively. Both were included as socioeconomic covariates in the fully adjusted model (Model 3) of Tables 2 and 3.

Abbreviations: JPY, Japanese yen; SD, standard deviation.

### 3.2 Associations Between Social Contact and Poor Mental Health

Fully adjusted results are detailed in Tables 2 and 3. For non‐cohabiting family/relatives, in‐person contact was associated with lower odds of poor mental health in a graded manner, with odds ratios of 0.84 (95% CI 0.71–0.99) for 1–3 times/month and 0.71 (0.57–0.89) for ≥ 1 time/week compared with none, and a significant trend across categories (*p* for trend = 0.003). Weekly phone contact suggested a modest association with lower odds (OR 0.81, 95% CI 0.65–1.01), while email/messages and video contact were not clearly associated after full adjustment.

**TABLE 2:** Association between frequency of social interaction with non‐cohabiting family/relatives and poor mental health (*N* = 6786).

Social interaction mode	Frequency	Model 1 (Unadjusted)		Model 2 (Demographic‐adjusted)		Model 3 (Fully‐adjusted)		*p* for trend
		OR (95% CI)	*p*	OR (95% CI)	*p*	OR (95% CI)	*p*	
In‐person	None	1.00		1.00		1.00		
	1–3	0.865 (0.736–1.017)	0.079	0.823 (0.698–0.971)	0.021	**0.835 (0.707–0.986)**	**0.034**	**0.003**
	≥ 1	0.774 (0.624–0.960)	0.020	0.705 (0.566–0.878)	0.002	**0.710 (0.570–0.886)**	**0.002**	
Phone	None	1.00		1.00		1.00		
	1–3	1.022 (0.866–1.206)	0.797	0.966 (0.816–1.143)	0.687	0.979 (0.826–1.161)	0.809	**0.041**
	≥ 1	0.905 (0.730–1.121)	0.353	0.799 (0.643–0.992)	0.043	0.810 (0.650–1.008)	0.059	
Email/messages	None	1.00		1.00		1.00		
	1–3	0.999 (0.824–1.212)	0.993	0.917 (0.753–1.118)	0.393	0.926 (0.759–1.131)	0.453	0.058
	≥ 1	0.961 (0.799–1.154)	0.664	0.818 (0.678–0.987)	0.036	0.848 (0.701–1.026)	0.090	
Video call	None (Reference)	1.00		1.00		1.00		
	1–3 times/month	0.929 (0.747–1.154)	0.503	0.902 (0.724–1.123)	0.355	0.906 (0.727–1.130)	0.383	0.235
	≥ 1 time/week	0.846 (0.586–1.220)	0.370	0.806 (0.557–1.165)	0.251	0.821 (0.567–1.190)	0.299	

*Note:* Multiple logistic regression was used. Model 1 was unadjusted. Model 2 adjusted for age, sex, household size, and morbidity. Model 3 additionally adjusted for smoking, drinking, walking, running/jogging, sport, working status, education, and household income. Education and household income were each entered as a binary indicator: junior college or lower versus all other responses, and < 4 million JPY versus all other responses, respectively. The reference category for each mode is “None.” *p* for trend tests linear trend across ordered categories (None < 1–3 times/month < ≥ 1 time/week) in Model 3. Estimates are unweighted. There were no missing values in the analytic variables among eligible older respondents, including education and household income. Multiplicity was addressed using Benjamini–Hochberg false discovery rate control across the 16 prespecified primary contrasts, as shown in Table S2; *p* for trend values are not FDR‐adjusted. Boldface indicates *p* < 0.05 before false discovery rate adjustment in Model 3.

Abbreviations: CI, confidence interval; FDR, false discovery rate; JPY, Japanese yen; OR, odds ratio.

**TABLE 3:** Association between frequency of social interaction with friends/acquaintances and poor mental health (*N* = 6786).

Social interaction mode	Frequency	Model 1 (Unadjusted)		Model 2 (Demographic‐adjusted)		Model 3 (Fully‐adjusted)		*p* for trend
		OR (95% CI)	*p*	OR (95% CI)	*p*	OR (95% CI)	*p*	
In‐person	None	1.00		1.00		1.00		
	1–3	0.727 (0.613–0.864)	< 0.001	0.703 (0.591–0.835)	< 0.001	**0.706 (0.593–0.842)**	**< 0.001**	**< 0.001**
	≥ 1	0.630 (0.510–0.779)	< 0.001	0.572 (0.467–0.702)	< 0.001	**0.592 (0.479–0.732)**	**< 0.001**	
Phone	None	1.00		1.00		1.00		
	1–3	1.101 (0.938–1.293)	0.235	1.065 (0.904–1.255)	0.451	1.079 (0.914–1.273)	0.369	0.845
	≥ 1	1.047 (0.837–1.309)	0.685	0.981 (0.780–1.236)	0.813	1.005 (0.796–1.270)	0.967	
Email/messages	None	1.00		1.00		1.00		
	1–3	1.047 (0.875–1.252)	0.616	0.954 (0.793–1.146)	0.612	0.976 (0.810–1.176)	0.780	0.168
	≥ 1	0.982 (0.816–1.182)	0.846	0.856 (0.707–1.035)	0.109	0.902 (0.740–1.098)	0.303	
Video call	None (Reference)	1.00		1.00		1.00		
	1–3 times/month	1.225 (0.862–1.741)	0.259	1.246 (0.876–1.772)	0.222	1.217 (0.853–1.738)	0.279	0.312
	≥ 1 time/week	0.843 (0.434–1.638)	0.615	0.668 (0.334–1.333)	0.252	0.668 (0.333–1.342)	0.257	

*Note:* Multiple logistic regression was used. Model 1 was unadjusted. Model 2 adjusted for age, sex, household size, and morbidity. Model 3 additionally adjusted for smoking, drinking, walking, running/jogging, sport, working status, education, and household income. Education and household income were each entered as a binary indicator: junior college or lower versus all other responses, and < 4 million JPY versus all other responses, respectively. The reference category for each mode is “None.” *p* for trend tests linear trend across ordered categories (None < 1–3 times/month < ≥ 1 time/week) in Model 3. Estimates are unweighted. There were no missing values in the analytic variables among eligible older respondents, including education and household income. Multiplicity was addressed using Benjamini–Hochberg false discovery rate control across the 16 prespecified primary contrasts, as shown in Table S2; *p* for trend values are not FDR‐adjusted. Boldface indicates *p* < 0.05 before false discovery rate adjustment in Model 3.

Abbreviations: CI, confidence interval; FDR, false discovery rate; JPY, Japanese yen; OR, odds ratio.

For friends/acquaintances, in‐person contact showed the strongest and most consistent associations. Compared with no in‐person contact, 1–3 times/month was associated with an odds ratio of 0.71 (95% CI 0.59–0.84) and ≥ 1 time/week with 0.59 (0.48–0.73), with a strong monotonic trend (*p* for trend < 0.001). Phone, email/messages, and video contact with friends were not associated with poor mental health after adjustment.

After applying Benjamini–Hochberg false discovery rate control across the 16 prespecified primary contrasts, the inverse associations for weekly in‐person contact with non‐cohabiting family/relatives (FDR‐adjusted *p* = 0.011) and for both monthly and weekly in‐person contact with friends/acquaintances (both FDR‐adjusted *p* < 0.008) remained statistically significant, whereas suggestive findings for remote modes did not survive correction (Table S2).

## 4 Discussion

In this nationwide web‐based sample of older adults in Japan, more frequent face‐to‐face contact was consistently associated with lower odds of poor mental health, defined as reporting at least one poor mental health day in the past 30 days, even after additional adjustment for education and household income. This pattern was most pronounced for in‐person contact with friends/acquaintances, and it was also evident for in‐person contact with non‐cohabiting family/relatives. In contrast, most remote contact modes were not clearly associated with poor mental health after adjustment, although weekly phone contact with non‐cohabiting family/relatives showed a modest borderline association.

These findings are consistent with conceptual frameworks and empirical evidence linking social integration, social support, and social ties to mental and physical health [1, 2, 13]. Face‐to‐face encounters may provide interpersonal features that are difficult to replicate remotely, including richer nonverbal cues, shared attention, emotional immediacy, and opportunities for shared activities. Such features may buffer stress, support positive affect, maintain behavioral routines, and strengthen perceived belonging [3, 4, 5, 6, 13, 14, 15]. The stronger associations observed for contact with friends may reflect the discretionary and activity‐oriented nature of many friendships in later life. Longitudinal evidence suggests that social connections can predict subsequent subjective wellbeing among older adults [14], and social identity perspectives emphasize that group‐based participation can support health by strengthening belonging and meaning [15]. In this context, routine in‐person contact with friends may function not only as emotional support but also as a marker of ongoing social participation, which is a core element of healthy aging.

The Japanese context is important for interpreting these findings. Japan has a rapidly aging population, an increasing number of older adults living alone, and strong policy interest in community‐based prevention and healthy aging. In this context, face‐to‐face contact with friends/acquaintances may represent more than simple communication; it may indicate participation in local groups, hobbies, walking, volunteering, or other discretionary activities that support identity, routine, and belonging. These mechanisms may help explain why associations were particularly strong for in‐person contact with friends/acquaintances.

Our results also align with prior evidence suggesting that the mode of contact matters. In a US cohort, face‐to‐face contact, but not phone or email, predicted lower subsequent depression in older adults [16]. While our study differs in setting and outcome, the convergence supports the possibility that in‐person interaction is particularly relevant for mental well‐being in older age. The largely null pattern for email/messages and video contact is compatible with systematic reviews showing heterogeneous and often modest effects of digital interventions on loneliness and mental health outcomes [10, 11, 12]. Remote contact may vary in purpose, depth, and emotional salience. Asynchronous messaging in particular may serve practical coordination rather than emotionally meaningful connection. In contrast, phone calls are synchronous and voice‐based, potentially conveying emotional nuance with relatively low technological burden; this may partly explain why weekly family phone contact suggested a modest association when in‐person contact is difficult.

From a geriatric practice and planning perspective, the present findings highlight potentially actionable targets that are simple, low‐cost, and scalable. Facilitating safe, routine face‐to‐face engagement, especially with friends/acquaintances, may be a practical avenue to support mental well‐being as part of comprehensive healthy aging strategies. Community‐based opportunities such as peer clubs, hobby circles, walking groups, volunteering activities, and accessible community spaces may help maintain social participation and thereby support mental well‐being and functional ability. When in‐person contact is not feasible, structured weekly phone calls with family members may be a pragmatic complement, particularly when supported by care managers, community nurses, family caregivers, local volunteers, or municipality‐based programmes.

Several limitations should be considered. First, the cross‐sectional design precludes causal inference and allows reverse causation, such that poor mental health may reduce motivation or opportunity for in‐person contact. We therefore interpret the findings as associations rather than causal effects and anticipate future work using longitudinal information from JACSIS as additional waves become available [18].

Second, the web‐based data collection method and the use of a non‐probability commercial online panel require careful interpretation in relation to the digital divide and representativeness. Although sampling was designed to reflect the distributions of sex, age groups, and residential regions, older adults without internet access, with low digital literacy, or with cognitive or functional limitations may have been less likely to participate. Such individuals may also have different patterns of social contact and poorer mental health. In addition, upstream panel‐management information, including the number invited, the number completing the survey before quality control, the response rate, and the number excluded by the quality‐control procedures, was not available in the analysis‐ready dataset provided to the authors, which limits full assessment of participation‐related selection. Furthermore, although the required‐response format minimized missing data, it may have introduced response bias if some participants provided less‐considered answers in order to complete the survey, particularly for sensitive items such as mental health. Therefore, the analytic sample may under‐represent the most socially or digitally excluded older adults, and the findings should not be interpreted as nationally representative prevalence estimates.

Third, the analyses were unweighted. Although the JACSIS survey used a large nationwide internet survey panel with sampling design and quality‐control procedures, the present analysis was based on an older‐adult analytic subset, and individual‐level sampling weights appropriate for weighted regression within this specific subset were not available. If participation in the internet panel or survey response was related to both social contact and mental health, selection bias may have affected the estimated associations.

Fourth, residual confounding is possible. Although the models adjusted for age, sex, household size, morbidity, lifestyle factors, physical activity, working status, education, and household income, socioeconomic position was captured using binary indicators, and unmeasured or more granular socioeconomic and social‐contextual factors—such as lifetime occupational position, household assets, and neighborhood socioeconomic context—may still influence both opportunities for social contact and mental health. Marital status was not included in the primary model and may shape the meaning and availability of family and friend contact. Direct measures of functional ability, ADL, IADL, or mobility limitation were also not available in the analytic dataset. Therefore, residual confounding by marital, functional, and other social‐contextual factors cannot be excluded.

Fifth, we modeled each exposure separately; because social contact across partners and modes is correlated, estimates should not be interpreted as independent effects of one mode holding all others constant. We also did not conduct extensive post hoc interaction analyses because of model complexity and multiplicity across partner–mode exposures. Future studies should examine whether marital status, living arrangement, availability of family contact, and low in‐person contact modify the association between remote contact and mental health.

Sixth, the binary outcome of any versus no poor mental health day was selected based on the response distribution and provides a sensitive indicator of any recent poor mental health, but it does not represent a clinical diagnosis or screening threshold and does not capture severity gradients such as frequent mental distress. Finally, video contact was rare, limiting precision.

Despite these limitations, the study's strengths include a large nationwide web‐based sample of older adults, explicit separation of interaction partner and contact mode, graded associations for in‐person contact, and prespecified control of the false discovery rate across primary contrasts [17].

## 5 Conclusions

Among older adults in Japan, frequent in‐person contact with friends/acquaintances and with non‐cohabiting family/relatives was associated with lower odds of poor mental health, whereas most remote contact modes were not clearly associated after full adjustment, including education and household income, and after multiplicity control. Facilitating regular face‐to‐face engagement, complemented by scheduled family phone calls when in‐person contact is difficult, may support mental well‐being in later life.

## Author Contributions

**Kazumi Kubota**, **Miya Aishima**, **Yukiko Matsumura:** conceptualisation. **Kazumi Kubota**, **Takahiro Tabuchi:** methodology. **Kazumi Kubota**, **Takeshi Miura:** formal analysis. **Takahiro Tabuchi:** data curation. **Kazumi Kubota:** writing – original draft. **Kazumi Kubota**, **Miya Aishima**, **Yukiko Matsumura**, **Takeshi Miura**, **Takahiro Tabuchi:** writing – review and editing. **Takahiro Tabuchi:** supervision. **Takahiro Tabuchi:** funding acquisition. All authors approved the final manuscript and agree to be accountable for all aspects of the work.

## Funding

This study (JACSIS 2024) was supported by the Japan Society for the Promotion of Science (JSPS) KAKENHI Grants (JP21H04856; JP25H01079 [Dr Takahiro Tabuchi]; JP24K15576 [Dr Kanami Tsuno]); JST RISTEX (JPMJRX21K6 [Dr Midori Matsushima]); the intramural fund of the National Institute for Environmental Studies (Dr Shoji Nakayama); the Health Labor Sciences Research Grants (23EA1003 [Dr Masayoshi Zaitsu]; 22FA2001 [Dr Daisuke Nishi]; 24LA0201 [Dr Atsushi Nakagomi]; 22JA1005; 22FA1001; 22FA1010; 23EA1001; 23FA1004; 24FA1020; 24LA1002 [Dr Takahiro Tabuchi]); and the Japan Agency for Medical Research and Development, Strategic Center of Biomedical Advanced Vaccine Research and Development for Preparedness and Response (JP243fa627004 [Dr Yuki Furuse]). The funders had no role in study design; data collection, analysis, or interpretation; manuscript writing; or the decision to submit the article.

## Disclosure

The authors have nothing to report.

## Ethics Statement

This study used de‐identified data. Ethical approval was obtained from the Shimonoseki City University Research Ethics Committee (acceptance no. 2509‐0623) and the Tohoku University Graduate School of Medicine Ethics Committee (rapid review dated 26 March 2025; study number 2024‐1‐1035). Participants provided electronic informed consent. The study was conducted in accordance with the Declaration of Helsinki and relevant institutional guidelines and regulations.

## Conflicts of Interest

The authors declare no competing interests related to this work. Dr. Tabuchi received financial support for research from Daiichi Sankyo Healthcare Co. Ltd., Johnson & Johnson K.K., Data Seed Inc., Workout‐Plus LLC, EMMA Co. Ltd., Addness Inc., and CotoIT Inc. (in the last 36 months). These entities were not involved in the study design; data collection, analysis, or interpretation; manuscript writing; or the decision to submit the article.

## Supporting information


**Table S1:** Distribution of social interaction frequency across the eight partner–mode exposures.
**Table S2:** Primary contrasts with nominal and false discovery rate‐adjusted *p*‐values in the fully adjusted model.

## Data Availability

The dataset is not publicly available due to governance agreements for specially processed, de‐identified information and participant privacy. Data may be available from the JACSIS steering group upon reasonable request and with necessary approvals. Further information is available from the JACSIS study website (https://jacsis‐study.jp/), and initial inquiries should be directed to Dr. Takahiro Tabuchi (tabuchitak@gmail.com).
